# Mesenchymal Stem Cell Secretome in Tendon Regeneration: Therapeutic Potential, Mechanisms of Action, and Future Perspectives

**DOI:** 10.3390/cells15090815

**Published:** 2026-04-30

**Authors:** Tatiana D. Dias, David E. Anderson

**Affiliations:** Large Animal Clinical Sciences Department, College of Veterinary Medicine, University of Tennessee, Knoxville, TN 37996, USA

**Keywords:** tendon healing, tendon injuries, mesenchymal stem cells, secretome, conditioned media, extracellular vesicles

## Abstract

**Highlights:**

**What are the main findings?**

- Mesenchymal stem cell (MSC) secretome derivatives, including conditioned media and extracellular vesicles, promote tendon healing primarily through paracrine mechanisms that modulate inflammation, enhance extracellular matrix remodeling, and support tenogenic regeneration.
- Preclinical evidence demonstrates consistent improvements in tendon structure, collagen organization, and biomechanical properties following MSC secretome treatment, although variability in secretome composition and experimental design remains significant.

**What are the implications of the main findings?**

- MSC secretome represents a promising cell-free therapeutic alternative to stem cell-based approaches, offering advantages in safety, scalability, and regulatory feasibility for tendon repair.
- Standardization of secretome production, improved functional outcome measures, and robust large animal and clinical studies are critical to enable successful translation into clinical practice.

**Abstract:**

Tendon injuries are common and debilitating musculoskeletal conditions that impose pain and debilitation to patients, significant challenges to medical professionals, and financial burdens to the healthcare system. Due to limited natural healing capacity, tendons typically undergo scar-mediated repair that compromises biomechanical integrity and increases the risk of reinjury. Despite a variety of therapeutic strategies, functional tendon healing remains a major clinical challenge. Mesenchymal stem cells (MSCs) represent an attractive strategy to improve tendon healing, largely due to their immunomodulatory and regenerative properties. Increasing evidence suggests that the therapeutic potential of MSCs is primarily attributed to their paracrine activity via the release of the secretome, a set of bioactive molecules that are known to mimic the immunomodulatory and regenerative properties of their parental cells. More recently, acellular approaches using MSC secretome derivatives, such as conditioned media and extracellular vesicles, have been largely explored for tendon healing. This review of the literature explores the therapeutic potential of MSC secretome derivatives for tendon healing, highlighting their advantages over cell-based therapies, proposed mechanisms of action, manufacturing and scalability considerations, and current state of research.

## 1. Introduction

Tendons are well-organized connective tissues attaching muscles to the skeleton. They play an important role in transmitting high contraction forces generated by muscle to bone to maintain posture and produce joint movement [1,2,3,4,5,6]. Tendons consist primarily of densely packed collagen fibers and tendon-specific cell types, tenocytes and tenoblasts, embedded in an extracellular matrix (ECM) [2,4,7,8]. The ECM is the non-cellular component, composed of collagen fibers, primarily collagen type I, and other non-collagenous proteins, predominantly proteoglycans, glycoproteins and glycosaminoglycans [2,9,10,11,12]. Those components provide physical support to the collagen fibers, essential to ensuring the mechanical function of the tendon, and maintaining a microenvironment crucial for tissue homeostasis, differentiation, and morphogenesis [9,10,13]. Collagen, synthesized by tenocytes, forms the fundamental force-transmitting unit of the tendon. They are aligned with the muscle–bone axis to form a collagen structure with unique biomechanical properties that can resist tensile and compressive forces [2,4,14]. However, overuse or mechanical loading above the biomechanical limit of the tissue commonly results in injuries, such as rupture, tear, or degeneration of the tendon [2,15,16,17].

Tendon injuries are debilitating musculoskeletal disorders that commonly affect human and veterinary patients [16,18,19]. These injuries are characterized by localized pain, swelling of the tendon, loss of function, and impaired performance [17,18,20,21]. They also represent significant financial hardship and medical challenges [7,22]. In the United States alone, musculoskeletal injuries account for an estimated $30 billion annually, with 45% to 55% involving tendons and ligaments [2,23,24].

Natural tendon healing is a lengthy process and often yields suboptimal structural, mechanical and functional outcomes [7]. Limited and inefficient intrinsic healing capacity, owing to relative hypocellularity and hypovascularity of the tissue, impairs restoration of an organized ECM [25,26]. Despite intensive remodeling after injury, the naturally healed tendon appears scar-like and does not completely recover its biomechanical properties [27,28,29,30]. This results in a healed tendon with reduced tensile strength and increased risk of reinjury [29,31].

Current treatment options for tendon injuries depend on the severity of the lesion and are generally limited to either conservative management or surgical intervention [14]. Mild-to-moderate injuries that do not significantly impair functions are typically treated conservatively. However, conservative approaches often show limited success in severe cases, such as complete tendon ruptures, making surgery necessary. Surgical management is also recommended when conservative treatment fails [14]. Despite their widespread use, both conservative and surgical treatments are associated with suboptimal long-term outcomes, including prolonged healing times, excessive scar tissue formation, and high reinjury rates [26].

To improve outcomes, several strategies have been explored, including stem cell/stromal cell therapy, platelet-rich plasma (PRP), and biomaterials, alone or seeded with stem cells [7,32]. PRP is an autologous, blood-derived therapy that represents a clinically established source of bioactive factors with secretome-like activity. It contains platelets, variable numbers of white blood cells, a complex mixture of growth factors and other bioactive molecules, including chemokines, cytokines, proteinases, clotting factors, and adhesive proteins [33]. Despite its widespread clinical use in tendon disorders, the reported benefits of PRP remain controversial, largely due to heterogeneity in its composition and lack of standardized preparation protocols, which directly influence its biological activity [7].

Despite the development of multiple therapeutic strategies, functional tendon healing continues to be a major clinical challenge [7,34,35]. To date, no available therapy can fully restore the native structural and mechanical properties of injured tendons [36,37]. In this context, the therapeutic potential of mesenchymal stem cell (MSC) secretome derivatives, such as conditioned media (CM) and extracellular vesicles (EVs), have emerged as promising acellular therapeutic approaches. These derivatives have the potential to modulate inflammation, promote matrix remodeling, and enhance tenogenic repair. However, important questions remain regarding their long-term safety and efficacy, mechanisms of actions, optimal formulation, and dosing strategies.

This review provides a comprehensive overview of MSC secretome derivatives in tendon healing, with a focus on their biological effects, proposed mechanisms of action, and translational challenges. Further, we discuss key considerations related to manufacturing, standardization, and scalability, and evaluate the current preclinical and emerging clinical evidence.

## 2. Literature Search Strategy

This narrative was conducted using a structured literature search to identify relevant studies on MSC-derived secretome for tendon healing. Databases including PubMed, Scopus and Web of Science were searched using combinations of the following keywords: “mesenchymal stem cells”, “secretome”, “conditioned media”, “extracellular vesicles”, “exosomes”, and “tendon healing”. Studies were selected based on relevance to tendon biology, regenerative mechanisms, and preclinical or clinical applications. Priority was given to recent studies and those providing mechanistic or functional insights into tendon healing. Reference lists of selected articles were also screened to identify additional relevant studies.

## 3. Role of Mesenchymal Stem Cells in Tendon Regeneration

Among stem cell types explored for regenerative medicine, MSCs are of particular interest [19]. MSCs are multipotent progenitor cells capable of self-renew and differentiation into multiple lineages [19,38]. In addition to these properties, their paracrine activity plays a central role in their therapeutic effects. MSCs secrete a complex set of soluble bioactive molecules, collectively referred to as the secretome, which includes growth factors, cytokines, and chemokines, and extracellular vesicles (EVs) [39]. These components are present in the conditioned media (CM) in which MSCs are cultured [39,40].

MSC-based therapy represents an attractive approach for tendon repair in human and veterinary sports medicine due to its ability to modulate inflammation and promote tissue regeneration [41]. In veterinary medicine, intralesional implantation of MSCs for equine tendonitis was first reported in 2003 [42], and has since been widely used for musculoskeletal injuries, particularly tendon and ligament disorders [43]. Clinical and experimental studies in horses have demonstrated improved tendon healing and reduced reinjury rates following MSC treatment [44,45,46,47]. The horse represents the only available animal model in which spontaneous, naturally occurring large tendon injury occurs [48,49], and the equine superficial digital flexor tendon (SDFT) is analogous to the human Achilles tendon in terms of function and injury susceptibility. This provides a valuable large animal model for studying tendon injury and repair in humans.

Despite promising veterinary and preclinical outcomes, translation of MSC-based therapies to human clinical use remains largely limited [36,50]. Challenges include MSC heterogeneity, regulatory constraints, lack of standardized isolation and expansion protocols, and unresolved safety concerns. Therefore, surgery and conservative treatment combined with physical therapy remain the standard of care for tendon injuries in humans [51].

Bone marrow-derived MSCs (BMSCs) are the most extensively studied and well-characterized, and remain the primary cell source for tendon regeneration strategies [41,51]. Adipose tissue-derived MSCs (ASCs) have also gained attention due to their accessibility, minimally invasive harvest, and lower patient morbidity [52]. More recently, tendon stem cells (TSCs) have been identified [53] and explored as a promising alternative for tendon healing [52,54,55,56]. These cells represent 1–4% of nucleated tendon cells and share key MSC characteristics, including self-renewal, trilineage differentiation, and surface marker expression [53,57]. Notably, TSCs can differentiate into tenocytes and form tendon-like tissue in vitro and in vivo [58,59,60], and have demonstrated superior regenerative potential compared to BMSCs in preclinical models [61]. These findings suggest that the choice of MSC source may influence therapeutic outcomes, highlighting the need for further comparative and translational studies.

## 4. MSC-Derived Secretome as a New Regenerative Approach

The direct replacement of necrotic or apoptotic cells through local engraftment and differentiation into tissue-specific lineages was initially proposed as the primary therapeutic mechanism of MSCs [62,63,64,65]. Supporting this, studies using labeled MSCs demonstrated their ability to migrate to sites of injury after in vivo administration, with some evidence of differentiation [66,67,68]. In vitro cell culture studies further suggested that MSCs can differentiate into non-mesenchymal cells, including neural cells, cardiomyocytes, hepatocytes, and endothelial cells under appropriate experimental conditions [69,70,71,72].

However, several in vivo studies reported functional improvements despite minimal evidence of MSC engraftment or differentiation, suggesting limited grafting potential and short-term survival following transplantation. For example, in a mouse model of myocardial infarction, systemic administration of human MSCs (hMSCs) improved cardiac function without detectable engraftment after three weeks [73]. Similarly, in children with osteogenesis imperfecta, MSC therapy resulted in significant clinical improvement despite less than 1% of donor cells being detected in recipient tissues four to six weeks post-infusion [74].

These findings have shifted the understanding of MSC fate after in vivo transplantation and mechanisms of action. Accumulating evidence suggests that their therapeutic benefits are primarily mediated through paracrine signaling via the release of the secretome, rather than long-term engraftment or differentiation [39,75,76,77,78,79]. Accordingly, the term “mesenchymal stem cells” has been reconsidered as “medicinal signaling cells” to more accurately reflect their mode of action [80]. Supporting this concept, CM from MSC cultures contains a rich repertoire of bioactive proteins and EVs that mirror many of the immunomodulatory and regenerative effects of their parental cells, both in vitro and in vivo.

### 4.1. MSC-Derived Conditioned Media (CM)

**Figure 1:**
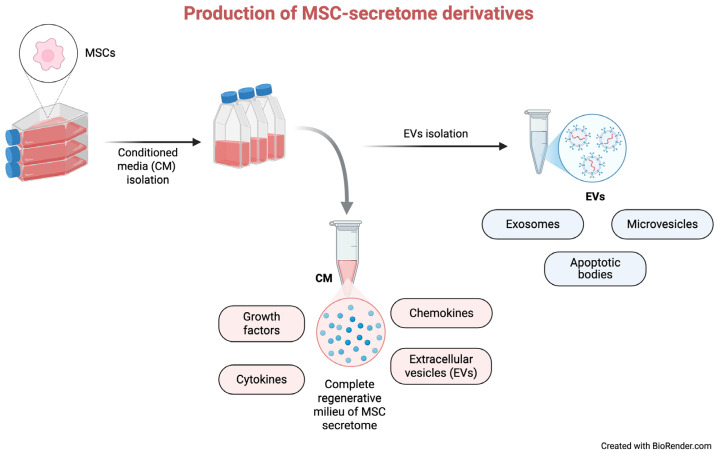
Production of MSC secretome derivatives and their main components. MSCs are cultured in vitro to generate CM, which contains the full spectrum of secreted bioactive factors. Following CM collection, the complete secretome can be used directly or further processed to isolate EVs. This schematic highlights the relationship between CM and EV fraction and illustrates how different secretome derivatives can be obtained for therapeutic applications.

Conditioned media (CM) refers to the culture media in which MSCs have been cultured and contains the complete regenerative milieu of MSC secretome, including soluble proteins and EVs [39,40] (Figure 1). Both MSC-CM and isolated EVs have been shown to promote tissue regeneration in a variety of diseases, including skin wounds, lung and liver injury, uveitis, myocardial infarction, renal disease, and musculoskeletal conditions such as tendon and ligament injuries [54,81,82,83,84,85,86,87,88,89,90,91].

In tendon models, TSC-derived CM improved Achilles tendon healing in rats by modulating ECM composition, promoting angiogenesis, enhancing collagen fiber alignment, reducing inflammation, and improving biomechanical properties [54]. Similarly, ASC-derived EVs promoted tendon healing in a rabbit Achilles tendon model, enhancing ECM remodeling, mechanical strength, and collagen organization while attenuating inflammation [92].

As the need grows for therapies that enhance tendon healing and restore function, both MSC-CM and EVs have emerged as promising cell-free alternatives. Their therapeutic potential is largely attributed to their immunomodulatory and regenerative properties, which will be explored in detail later in this review. While both MSC-CM and EVs have demonstrated regenerative potential, their relative contribution to tendon healing remains incompletely understood. Increasing evidence suggests that the therapeutic effects of the secretome may result from the synergistic interaction between soluble factors and vesicular cargo. In this context, complete CM may provide broader biological activity compared to isolated EVs, particularly in inflammatory environments where multiple signaling pathways are involved. However, EV-based approaches may offer advantages in terms of product standardization and scalability, which are critical considerations for clinical translation.

### 4.2. Extracellular Vesicles (EVs)

Extracellular vesicles (EVs) are heterogeneous, nano-sized, membrane-bound particles actively released by nearly all cell types into the extracellular environment via exocytosis. They consist of a phospholipid bilayer derived from the parent cell and enclose a variety of bioactive cargos, including proteins, lipids, and nucleic acids [93,94]. EVs are broadly categorized based on size and biogenesis into exosomes (~40–160 nm), microvesicles (~100–1000 nm), and apoptotic bodies (>1000 nm). Among these, exosomes and microvesicles are the most extensively studied and are commonly grouped under the term EVs. They can be distinguished by their size, molecular content, biological function, and mechanism of formation [39,93,95,96].

Recently, the therapeutic potential of secreted exosomes has received extensive attention. Their ability to transfer bioactive molecules from parent to recipient cells is of great interest for their potential value in clinical treatments [93,97,98]. This enables intercellular communication, improves cell adhesion and migration, and facilitates the exchange of genetic information between cells [93,95,96,97,99]. Exosomes have been isolated from CM and a variety of body fluids, including blood, serum, saliva, breast milk, amniotic fluid, bronchoalveolar lavage fluid, synovial fluid, and urine [100,101,102,103,104,105,106]. Their cargo depends on the type of releasing cell and may include a variety of proteins, metabolites, nucleic acids, RNA, DNA, receptors, transcription factors, enzymes, lipids, and ECM proteins from their parent cells [93,95,107,108]. Through specific surface interactions, exosomes deliver these molecules to target cells to modulate cellular function and signaling pathways [109,110]. Their phospholipid bilayer also confers stability and permeability, facilitating cellular uptake [110].

While most cells are known to release exosomes, MSCs are known to release greater quantities of exosomes [109] with unique biological functions due to the presence of a specific protein subclass cargo [93]. These vesicles have been shown to promote tissue repair, regeneration, immunomodulation, and angiogenesis [109]. Compared to MSC-based therapies, exosomes offer advantages such as lower immunogenicity and greater stability [109], making them attractive candidates for clinical application. In tendon models, MSC exosomes have been reported to reduce the early inflammatory response, inhibit fibrosis, and regulate ECM remodeling, contributing to improved tendon healing [55,111,112]. Consequently, EV-based therapies are increasingly being explored as a cell-free approach for tendon regeneration.

### 4.3. Benefits of Cell-Free Therapy Compared with Stem Cell-Based Applications

There is increasing interest in MSC secretome derivatives due to safety concerns and limitations associated with the transplantation of living, proliferative cells [113]. Stem cell-based therapies carry risks including immune rejection, poor engraftment and survival, transmission of infections, tumorigenic potential, and undesired spontaneous differentiation [39,54,78,114,115]. For example, Liu et al. demonstrated that TSCs underwent erroneous differentiation toward chondrogenic and osteogenic lineages when exposed to degenerative tendon matrices in vitro, likely influenced by the pathological microenvironment [116].

In addition to safety concerns, stem cell therapies present significant logistical and biological challenges. These include variability in donor cell quality, lack of standardized production protocols, and the need for rigorous quality control to ensure therapeutic efficacy [50,117]. Autologous approaches require invasive cell harvesting, are time-consuming, and involve expensive cell expansion processes. Moreover, MSC quality and efficacy can be affected by exposure to inflammatory environments and donor-related factors, such as age and overall health [118,119,120,121,122]. For instance, inflammatory environments have been shown to impair the tenogenic potential of equine ASCs, suggesting that tenogenic differentiation of MSCs resulting in direct cell replacement is unlikely in acute tendon injury environments [49].

These limitations have driven interest in MSC secretome as a cell-free alternative that leverages the immunomodulatory and regenerative properties of MSCs without relying on live cells. MSC secretome-based therapies mitigate many of the risks associated with cell transplantation, including immunogenicity, tumorigenesis, infection, and undesirable spontaneous differentiation [39,123,124,125], representing a potentially safer alternative compared to cell-based therapies. In addition, their safety, potency, and dosage can be evaluated in a manner analogous to conventional pharmaceutical products [39].

From a practical standpoint, secretome derivatives offer important advantages. CM can be produced in large quantities, lyophilized, and stored long-term without losing bioactivity [126], eliminating the need for cryopreservatives present in cell freezing media such as DMSO [127]. For example, lyophilized CM from human uterine cervical stem cells has been shown to retain therapeutic efficacy in promoting corneal healing in rats after storage and reconstitution [126]. Furthermore, these therapies avoid invasive cell collection and complex culture procedures, reducing both treatment cost and time, and simplifying handling and administration [39,125]. In a clinical study in 13 horses with spontaneous tendon or ligament injuries, intralesional administration of MSC-CM was well-tolerated, with no adverse reactions, inflammation, or tumor formation reported, demonstrating its safety for intralesional administration [128].

Importantly, MSC secretome can be manufactured on a large scale using standardized cell lines under controlled conditions, improving reproducibility and consistency of bioactive composition [39]. Moreover, their low metabolic activity and acellular nature further facilitate quality control and regulatory compliance, supporting their development as pharmaceutical-like products [129,130]. In addition, MSC secretome can be freeze-dried, packaged, stored, and transported more easily than living cells [130], enabling off-the-shelf application [39,125,129,131]. The use of allogeneic sources from healthy, young donors further enhances scalability and accessibility while minimizing safety concerns [39,118,132].

### 4.4. MSC Secretome Production and Limitations

Although the regenerative potential of MSC secretome has been widely demonstrated, clinical translation in veterinary and human medicine requires standardized and reproducible manufacturing protocols. Such standardization is essential to minimize batch-to-batch variability, ensure product quality and safety, and achieve consistent therapeutic outcomes.

MSC-CM is typically produced by culturing cells for a defined period, followed by centrifugation and filtration to remove cellular debris and collect the supernatant containing the secretome [133,134]. However, several variables in this process remain unstandardized. Variations in basal media, cell confluency at the time of collection, and passage number can significantly influence the composition of CM [135,136]. In most studies, MSCs are cultured in serum-free conditions and CM is collected within 72 h of culture when cells are approximately 70–90% confluent [129]. The use of fetal bovine serum (FBS) is generally avoided due to risks of immunogenic reactions, potential contamination with endotoxins and viruses [137,138], and the introduction of FBS-derived growth factors and exosomes, which can compromise secretome purity and confound experimental outcomes [129]. Consequently, serum-free formulations are the current gold standard for MSC secretome production [138]. Defining culture parameters, including cell type, culture media, culture condition, conditioning time, passage number, number of cells, and processing methods, is therefore critical for establishing good manufacturing practices (GMPs) and enabling scalable production of MSC secretome products [129,131].

The EV fraction of MSC secretome can be isolated from CM of cultured cells [139]. However, manufacturing exosomes for therapeutic use involves several additional challenges, including variability in isolation, quantification, and characterization methods, inconsistent purity, and limited scalability [123,140,141]. The quantity and quality of exosomes released in culture may vary depending on several factors, including MSC source, culture conditions, and methods used for isolation [139,142,143,144]. The yield of exosomes is also inherently low, with MSCs typically releasing 1–4 μg of exosomes per 10^6^ cells over a 24 h culture period [143]. This has been a limitation for the use of exosomes in clinical trials, which requires quantities that often range between 40 and 50 mg per dose [145].

Traditional isolation methods include differential and ultracentrifugation, size-exclusion chromatography, immuno-based capturing, and precipitation-based methods [146]. However, most of these methods are time-consuming, require specialized equipment, and do not guarantee high yield or purity of exosomes [147]. Ultracentrifugation is the most widely used in academic research but is time-consuming and often results in incomplete sedimentation, aggregation of exosomes and contamination with cellular debris, proteins, and other EVs of different sizes [147,148,149]. Moreover, these traditional methods cannot be scaled up, hindering their applicability in clinical trials [150]. Emerging technologies, such as microfluidic-based systems, are being explored to enable rapid, effective, and clinically compliant exosome isolation [95,151].

A further challenge is exosome heterogeneity. The structure and cargo of exosomes depend heavily on the cell source, physiological state, and culture conditions, and even identical cell populations may present significant variability in cargo profiles under different conditions [93,152,153]. This variability contributes to batch-to-batch inconsistency and limits reproducibility across studies and therapeutic applications of EV-based products [154].

Taken together, these considerations highlight that variability in production methods and lack of standardization remain significant challenges that must be addressed to enable reproducibility, cross-study comparison, and successful clinical translation of MSC secretome therapies.

### 4.5. Scaling-Up Production of MSC Secretome

Advancing the clinical translation of MSC secretome requires scalable, cost-effective, and standardized manufacturing strategies. A major limitation to large-scale production is the finite proliferative capacity of MSCs, which undergo a limited number of divisions before entering senescence (the Hayflick limit) [39,155,156]. This results in the need for continuous isolation and expansion of new MSC batches, contributing to batch-to-batch variability and increased production costs [39,155].

To address this limitation, immortalized MSC lines have been investigated as a potential solution. For instance, Chen et. al. immortalized human embryonic stem cells (hESCs) via MYC oncogene transformation. Exosomes derived from these cells reduced infarct size in a murine myocardial ischemia/reperfusion model, indicating preserved therapeutic efficacy of exosomes. These findings support oncogene-mediated immortalization as a strategy to provide a stable and virtually unlimited MSC source for scalable secretome production [155].

In parallel, bioreactor-based culture systems also have emerged as an effective platform for large-scale MSC expansion and secretome production. Compared with conventional two-dimensional (2D) cultures, bioreactors enable controlled culture conditions, reduce the need for repeated passaging, and improve standardization and reproducibility of secretome output [39,157,158]. The integration of immortalized cell lines with bioreactor systems represents a promising strategy for scalable and standardized production of MSC secretome products [159].

Overall, these approaches demonstrate the feasibility of large-scale production, although further optimization and standardization will be essential to ensure consistent product quality and regulatory compliance.

### 4.6. Priming Strategies to Improve the Therapeutic Potential of MSC Secretome

**Figure 2:**
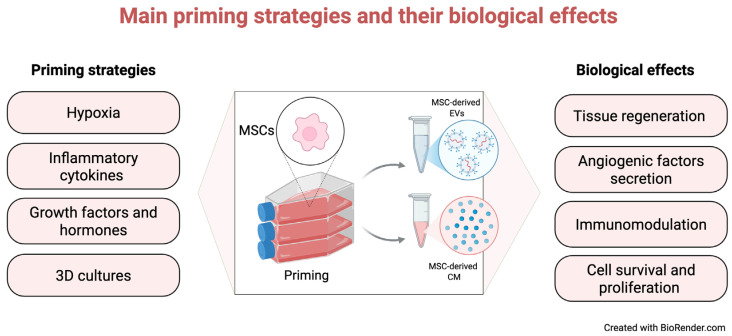
Main MSC priming strategies and their biological effects on MSC secretome and target tissue. MSCs can be preconditioned using different priming strategies, including hypoxia, inflammatory cytokines, growth factors and hormones, and 3D culture systems. These approaches modulate the secretory profile of MSCs, promoting key biological effects such as increased angiogenic factors secretion, immunomodulation, improved cell survival and proliferation, and overall enhancement of tissue repair processes.

The MSC secretome is a dynamic and context-dependent mixture of bioactive factors whose composition is strongly influenced by the surrounding microenvironment [160]. To harness and enhance its therapeutic potential, in vitro priming (preconditioning) strategies have been developed to modulate MSC secretory profiles for specific applications [39,50,160,161]. The main approaches include hypoxia, exposure to cytokines, growth factors, and hormones, and tri-dimensional (3D) culture systems (Figure 2).

Hypoxic preconditioning enhances the secretion of pro-angiogenic and regenerative factors [50] primarily through upregulation of hypoxia-inducible factor (HIF-1α), which increases expression of angiogenic mediators such as vascular endothelial-derived growth factor (VEGF) [162]. Consistently, increased VEGF and angiogenin levels have been reported in CM from hypoxia-primed MSCs, alongside enhanced migration and tube formation of human umbilical vein endothelial cells (HUVECs) [163]. Similarly, exosomes from hypoxia-stimulated BMSCs promoted in vitro HUVEC proliferation, migration, and angiogenesis [164]. Hypoxia also increases secretion of ECM remodeling factors, including matrix metallopeptidase (MMP)-2 and MMP-9 [165,166], and mediators of the inflammatory response such as IL-6 and IL-8 [167].

Priming with inflammatory cytokines enhances MSC immunomodulatory and regenerative properties by increasing the secretion of immune-regulatory factors [39,160]. For instance, human amnion-derived MSCs (hAMSCs) produced limited immunomodulatory factors under basal conditions [168], whereas interferon-gamma (IFN-γ) priming upregulated genes involved in the immune response, antigen presentation, and complement system activation [169]. Inflammatory cytokine priming also enhances the immunomodulatory properties of MSC-derived exosomes. For example, tumor necrosis factor-α (TNF-α)-primed MSCs showed increased anti-inflammatory effects and reduced endometrial fibrosis, primarily via exosome-mediated macrophage polarization toward an M2 phenotype [170].

Priming with growth factors and hormones enhances the regenerative and immunomodulatory properties of MSCs by favoring the secretion of tissue repair, angiogenesis and fibrosis inhibition factors [160]. For example, prostaglandin E2 (PGE2) priming reduced pro-inflammatory cytokines while increasing anti-inflammatory signaling and modulating macrophage polarization in an acute lung injury model [171]. Similarly, hepatocyte growth factor (HGF)-primed MSCs produced CM that suppressed inflammation, increased IL-10, and promoted angiogenesis and ECM remodeling through upregulation of MMP-2, MMP-9, and VEGF in a tendon injury model.

Compared to conventional 2D cultures, 3D culture systems better recapitulate the in vivo microenvironment and enhance MSC function by stimulating factors that favor cell survival and proliferation [39,160]. MSC spheroids increased the expression of CXCR4, improving cell adhesion, survival and homing [172], and enhanced the immunomodulatory and angiogenic properties of MSCs [172,173]. Further, CM derived from 3D-cultured hBMSCs showed markedly increased levels of IL-6, VEGF, IL-8, and chemokine ligand 1 (CXCL1) compared with CM from MSCs in 2D culture [174].

Collectively, these findings suggest that priming strategies represent a promising approach to enhance the therapeutic potential of MSC secretome. However, variability introduced by priming conditions highlights the need for careful standardization in future studies.

## 5. Potential Mechanisms of MSC Secretome on Tendon Healing

**Figure 3:**
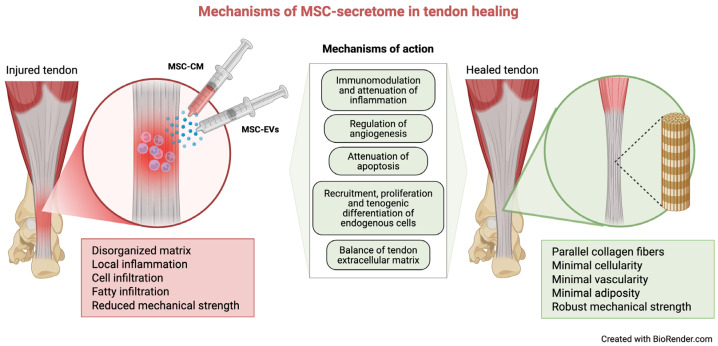
Mechanisms of MSC secretome in tendon healing. Following injury, tendons are characterized by a disorganized ECM, local inflammation, cellular and fatty infiltration, and reduced mechanical strength. Administration of MSC-CM or -EVs delivers a complex mixture of bioactive factors that modulate regenerative pathways. These include immunomodulation and attenuation of inflammation, regulation of angiogenesis, reduction in apoptosis, and stimulation of recruitment, proliferation, and tenogenic differentiation of endogenous cells, as well as restoration of extracellular matrix balance. Collectively, these effects promote the transition from a degenerative to a regenerative microenvironment, favoring tendon functional healing.

Understanding the mechanisms behind MSC secretome-mediated tendon healing is essential for clinical translation. The therapeutic effects arise from the combined action of soluble factors and EVs, which collectively mediate paracrine signaling to modulate inflammation and promote tissue regeneration [175]. Figure 3 summarizes the main mechanisms of MSC secretome in tendon healing.

### 5.1. Immunomodulation and Attenuation of the Inflammatory Response

MSC secretome modulates the immune response by suppressing pro-inflammatory cytokines and enhancing anti-inflammatory signaling, including IL-10 [111]. ASC exosomes significantly reduced IL-1β and IL-6 expression in human rotator cuff tendons [176], while MSC-CM decreased IL-6 and TNF-α in an IL-1β-induced tendinopathic model [177].

Inflammation following tendon injury is largely driven by macrophages that migrate to the injured site, contributing to adhesion formation during tendon repair [178,179]. While M1 macrophages drive early inflammation, M2 macrophages favor tissue regeneration [180,181]. MSC-derived exosomes have been shown to regulate macrophage polarization, playing an important role in modulating the early inflammatory process and improving healing [176,182,183,184]. For example, ASC exosomes improved chronic rotator cuff tendinopathy by inhibiting M1 polarization and increasing M2 polarization, which reduced inflammatory infiltration and collagen disorganization [182]. Similarly, MSC-EVs attenuated early tendon inflammation via inhibition of macrophage nuclear factor-κB (NF-κB) signaling [183]. Similar shifts toward M2 macrophages and reduced pro-inflammatory cytokines have been reported in multiple tendon injury models [55,185,186].

Together, these findings suggest that modulation of the inflammatory response is one of the central mechanisms through which MSC secretome supports tendon healing by creating a microenvironment that favors tissue repair rather than persistent degeneration.

### 5.2. Regulation of Angiogenesis

Angiogenesis plays an important role during early tendon healing, resulting in neovascularization. This allows the delivery of fibroblasts, inflammatory cells, oxygen, nutrients and soluble factors to the site of injury, while removing waste products [187,188,189]. MSC secretome contains multiple pro-angiogenic soluble factors, including monocyte chemoattractant protein-1 (MCP-1), IL-8, macrophage inflammatory protein (MIP)-1α, MIP-1β, monokine induced by IFN-γ (MIG), stromal cell-derived factor-1 (SDF-1), and VEGF [54,190,191].

CM derived from HGF-primed TSCs promoted angiogenesis in Achilles tendons via upregulation of VEGF [54], while BMSC exosomes promoted proliferation, migration, and tube formation of human umbilical vein endothelial cells (HUVECs] [164,185], as well as peri-tendinous angiogenesis in vivo [185]. These findings indicate that MSC secretome-mediated modulation of angiogenesis supports the early reparative phase of tendon healing by enhancing vascular ingrowth and facilitating the delivery of essential cellular and molecular components to the injured tissue.

### 5.3. Attenuation of Apoptosis

MSC secretome exerts anti-apoptotic effects that may prevent excessive cell loss following injury. MSC-EVs reduced inflammation and apoptotic cell accumulation in patellar tendons of rats after local administration [192], and MSC exosomes attenuated cell apoptosis after a tendon–bone reconstruction in mice [186]. Reduced expression of caspase 3, a marker of apoptotic cells, has also been reported in Achilles tendon injuries treated locally with TSC exosomes [55]. These findings suggest that the anti-apoptotic properties of MSC secretome contribute to improved cellular survival within injured tendon, thereby supporting a more favorable environment for structural repair and functional healing.

### 5.4. Repopulation of Damaged Tissue Through Recruitment, Proliferation and Tenogenic Differentiation of Endogenous Cells

Tenocytes and TSCs play a crucial role in tendon homeostasis, function, ECM remodeling, and repair [19,193]. TSC exosomes increased proliferation and migration of TSCs in vitro via activation of the TGF bSmad2/3 and ERK1/2 signaling pathways [194]. Similarly, BMSC exosomes promoted tendon healing by facilitating the proliferation, migration, and tenogenic differentiation of TSCs in vitro and the proliferation of local TSCs in vivo [195]. Moreover, TSC exosomes promoted early tendon healing via miR-144-3p-regulated tenocyte proliferation and migration [196] and were shown to be taken up by tenocytes and favor their proliferation and migration in a process that may be dependent on the activation of the PI3K/AKT and MAPK/ERK1/2 signaling pathways [55]. TSC-CM also promoted the viability, proliferation and migration of tenocytes [54,197].

Overall, these studies indicate that MSC secretome not only enhances resident cell survival and migration but may also promote a more tenogenic cellular phenotype that supports functional tendon regeneration.

### 5.5. Balance of Tendon Extracellular Matrix and Fibrosis Inhibition

ECM degradation typically occurs as a result of the inflammatory response that develops after tendon injury, delaying the repair process [198]. MSC secretome regulates ECM turnover by modulating matrix synthesis and degradation. In a recent study, TSC exosomes decreased matrix metalloproteinase-3 (MMP-3) and increased tissue inhibitor of metalloproteinase (TIMP)-3, collagen (COL) 1a1 and tenomodulin (TNMD) expression, improving collagen organization and biomechanics in rats with Achilles tendon tendinopathy [56]. Similarly, TSC exosomes increased COL1a1 and TIMP-1 and reduced matrix metalloproteinase-9 (MMP-9), resulting in a favorable COL1a1/COL3a1 ratio [55]. Comparable improvements in ECM organization and mechanical properties have been reported with HGF-primed TSC-CM [54].

MSC exosomes also limit the fibrotic healing response that typically develops after tendon injury via different mechanisms. TSC exosomes reduced fibrosis and improved tendon healing by significantly decreasing the expression of COX-2 after Achilles tendon injury [91]. Similarly, BMSC exosomes significantly inhibited the fibrogenic process in vitro and in vivo via the let-7a/Tgfbr1 axis [112].

Overall, regulation of ECM turnover is a critical component of MSC secretome-mediated tendon repair, helping to improve tissue organization while limiting excessive fibrotic remodeling.

## 6. Current Status and Future Perspectives in the Repair of Tendon Injuries Using MSC Secretome

MSC secretome has emerged as a promising therapeutic approach for tendon repair in both veterinary and human regenerative medicine. Preclinical animal studies (Table 1) consistently report favorable effects of MSC-derived CM and EVs on tendon healing. However, there is substantial variability across studies in MSC secretome source, dosage, frequency of administration, and mode of delivery for tendon repair [129]. The lack of standardization in these parameters limits the comparability of results and represents a major barrier to clinical translation.

In addition to variability in experimental design, a major limitation across studies is the heterogeneity of MSC secretome composition. Factors such as donor variability, tissue source, dosing, culture conditions, and priming strategies significantly influence the composition and biological activity of the secretome. This variability limits reproducibility and complicates comparisons across studies, representing a major barrier to both mechanistic and clinical translation.

As shown in Table 1, most studies rely on small animal tendon injury models, particularly rodent models, which have a faster rate of tendon healing than humans. Among large animals, the horse represents the only available animal model of naturally occurring tendon injury [48,49]. Nonetheless, only a single preclinical study has reported intralesional administration of MSC-CM in horses with spontaneous tendon or ligament injuries [128], and a single case report describes MSC-EV treatment in a 6-year-old Dutch Warmblood gelding diagnosed with suspensory ligament injury [199]. Additionally, a pilot study investigating the therapeutic potential of MSC-EVs for acute rotator cuff injury in sheep has been published [200]. Importantly, the limited number of large animal studies is further compounded by the lack of objective functional outcomes. While histological and imaging improvements are frequently reported, these do not necessarily translate into meaningful functional recovery. The incorporation of standardized functional assessments, such as objective gait analysis and biomechanical testing, is essential to better evaluate the translational relevance of MSC secretome therapies.

Most preclinical studies on MSC secretome for tendon healing lack objective functional assessments such as gait analysis. A recent study reported the use of a video-based gait analysis to assess tendon functional healing after treatment with ASC-EVs in mice with Achilles tendon injury. Gait analysis demonstrated that EVs reduced functional deficits after Achilles tendon healing [201]. Functional assessments are key to determine if MSC secretome treatment yields improvements in tendon function and should be considered in further animal studies.

The relative efficacy of the complete CM versus the EV fraction alone remains poorly understood. Evidence suggests that the immunomodulatory and regenerative effects of MSC secretome result from the synergistic action of EV cargo and soluble proteins, implying that complete CM may have greater therapeutic potential than the EV fraction alone [202,203,204]. Supporting this, a recent study showed that MSC-CM was more effective than EVs alone in modulating inflammation in tenocytes [36]. Similarly, studies in other tissue models, including skeletal muscle [202], osteoarthritis [203], and discogenic pain [204], have reported enhanced or more comprehensive biological effects when CM is used compared to isolated EVs, likely due to the combined action of soluble factors and vesicular components. Based on these findings, it is plausible that CM may be particularly advantageous in inflammatory or complex microenvironments, such as tendon injuries, where multiple signaling pathways are involved, whereas EVs alone may provide more limited or targeted effects. However, these observations remain context-dependent, and further comparative studies are needed to clarify these differences and identify components that are most critical to therapeutic efficacy in tendon healing. From a translational perspective, this highlights a potential trade-off between the broader biological activity of CM and the more defined composition, reproducibility, and scalability of EV-based products.

Despite promising preclinical data, clinical translation remains limited. To date, only one active clinical trial is evaluating MSC secretome for a tendon disorder: a phase 1/2 study at Shanghai Sixth People’s Hospital (NCT07111325) testing the safety and preliminary efficacy of human-induced pluripotent stem cell-derived extracellular vesicle (iEV) for lateral epicondylitis of the humerus. Currently, there are no tendon-specific phase II or III clinical trials evaluating MSC secretome therapies, and available clinical evidence is restricted to early-phase studies. In addition, there is considerable uncertainty regarding optimal dosing, frequency of administration, and delivery methods, which remains largely inconsistent across studies. These factors represent critical gaps that must be addressed before widespread clinical application can be achieved. More robust preclinical evidence, generated from large animal models, is a necessary first step toward clinical trials designed to rigorously assess the safety and efficacy of MSC secretome for tendon healing.

In parallel with MSC secretome approaches, platelet-derived therapies such as PRP and platelet lysates represent clinically established sources of bioactive factors with secretome-like activity [205,206,207]. These products contain a complex mixture of growth factors, cytokines, and other bioactive molecules, including extracellular vesicles [205,208] and have been widely used in tendon repair [206,209]. However, similar to MSC secretome, their therapeutic efficacy is influenced by variability in composition and lack of standardization [205,210]. Notably, these limitations parallel those observed with MSC secretome-based approaches. Direct comparisons between platelet-derived products and MSC secretome may provide valuable insights into the relative advantages and limitations of these approaches.

**Table 1:** Summary of preclinical studies evaluating the use of MSC secretome for tendon healing.

Secretome Type	Source	Delivery Method	Target Tissue/Animal Model	*n*	Dose/Regimen	Standardized Outcome Domains *	Key Findings/Trends	Ref.
CM	Horse amniotic membrane MSCs	Local injection	Horse (spontaneous tendon/ligament injury)	13	Not specified, single	Clinical, Imaging	Reduced reinjury rate (15.38%); improved clinical and ultrasound outcomes	[128]
CM	HGF-treated TSCs	Injection (2×/week)	Rat (Achilles tendon injury)	63	100 µL for 2 weeks	ECM, Histology, Inflammation, Angiogenesis, Biomechanics	Enhanced ECM remodeling, angiogenesis, collagen alignment; reduced inflammation and scarring; improved biomechanics	[54]
CM	TSC + ASC	Injection	Rat (Achilles tendon injury)	14	Single	ECM, Histology, Angiogenesis	Increased collagen I synthesis (PINP); limited structural improvement	[211]
EVs	ADSCs	Injection	Rabbit (Achilles tendon injury)	36	Not specified	Biomechanics, Histology	Improved mechanical strength and histology	[92]
EVs	BMSCs	Injection	Rat (patellar tendon injury)	16	2 × 10^10^ particles	Inflammation, ECM	Reduced inflammation; improved collagen organization	[192]
Exosomes	TSCs	Injection	Rat (Achilles tendon injury)	18	50 µg	ECM, Biomechanics	Reduced fibrosis; improved strength	[56]
EVs	BMSCs	Injection	Rat (Achilles tendon injury)	16	10–30 µg	Biomechanics	Dose-dependent improvement	[37]
EVs	MSCs	Injection	Mice (Achilles tendon injury)	32	10^10^ particles	Inflammation, ECM	Reduced rupture; enhanced collagen	[183]
Exosomes	TSCs	GelMA hydrogel	Rat (Achilles tendon injury)	54	10 µg	Inflammation, ECM	Reduced scarring; improved structure	[55]
Exosomes	BMSCs	Injection	Rat (patellar tendon injury)	52	100 µg	Cell proliferation, ECM	Improved ECM remodeling	[195]
Exosomes	UC-MSCs ± miR-29a	Injection	Rat (Achilles tendon injury)	90	100 µg	ECM	Engineered exosomes superior	[212]
Exosomes	ADSCs	GelMA hydrogel	Rat (patellar tendon injury)	63	200 µg	Signaling, ECM	Activated SMAD pathways	[213]
Exosomes	TSCs	HA scaffold	Rat (patellar tendon injury)	72	200 µg	Histology	Improved fiber organization	[196]
EVs	iPSC-MSCs	Injection	Rat (quadriceps tendon injury)	30	Not specified	Inflammation	Promoted M2 polarization	[214]
Exosomes	UC-MSCs	Injection	Rat (Achilles tendon injury)	18	Not specified	Molecular signaling	miR-27b pathway	[214]
EVs	TNF-α ADSCs	Collagen sheet	Mice (Achilles tendon injury)	32	10^9^–5 × 10^9^	Inflammation	Improved healing	[215]
EVs	TSCs	Decellularized tendon scaffold	Rat (Achilles tendon injury)	72	Not specified	ECM	Improved collagen deposition	[216]
EVs	TSCs ± GelMA	GelMA vs. PBS	Rat (Achilles tendon injury)	96	500 µg	Release kinetics	Sustained release improved repair	[217]

* Standardized outcome domains include: inflammation, extracellular matrix (ECM) remodeling, histology, biomechanics, cellular responses (e.g., proliferation, apoptosis, macrophage polarization), molecular signaling, and clinical/imaging outcomes.

## 7. Conclusions

Growing evidence supports the therapeutic potential of MSC secretome for tendon healing. However, several key challenges must be addressed before these therapies can be translated into clinical practice. These include the substantial variability in secretome composition, lack of standardized protocols and characterization, and limited availability of large animal studies with functional outcome measures.

In addition, the absence of advanced clinical trials highlights the need for more rigorous preclinical and translational research. Future studies should prioritize standardized methodologies, direct comparisons between CM, EVs, and platelet-derived products, and the incorporation of clinically relevant functional endpoints, such as objective gait analysis and biomechanical analysis.

While MSC secretome represents a promising cell-free therapeutic strategy, a more critical and systematic approach will be essential to fully realize its potential for tendon regeneration.

## Data Availability

No new data were created or analyzed in this study. Data sharing is not applicable to this article.

## Abbreviations

The following abbreviations are used in this manuscript:

ASCs	Adipose tissue-derived MSCs
BMSCs	Bone marrow-derived MSCs
CXCL1	Chemokine ligand 1
COL	Collagen
CM	Conditioned media
EVs	Extracellular vesicles
GMP	Good manufacturing practices
HGF	Hepatocyte growth factor
AMC-CM	Horse amniotic membrane-derived MSCs CM
hAMSCs	Human amnion-derived MSCs
hESCs	Human embryonic stem cells
hMSCs	Human MSCs
HUVECs	Human umbilical vein endothelial cells
HIF-1α	Hypoxia-inducible factor
IBC	Immuno-based capturing
iEV	Induced pluripotent stem cell-derived extracellular vesicle
IFN-γ	Interferon-gamma
IL	Interleukin
MIP	Macrophage inflammatory protein
MMP	Matrix metallopeptidase
MSCs	Mesenchymal stem cells
MCP-1	Monocyte chemoattractant protein-1
MIG	Monokine induced by IFN-γ
NF-κB	Nuclear factor-κB
PrP	Platelet-rich plasma
PGE2	Prostaglandin E2
SEC	Size-exclusion chromatography
SDF-1	Stromal cell-derived factor-1
SDFT	Superficial digital flexor tendon
TSCs	Tendon stem cells
TNMD	Tenomodulin
TIMP	Tissue inhibitor of metalloproteinase
TNF-α	Tumor necrosis factor-α
2D	Two-dimensional
VEGF	Vascular endothelial-derived growth factor
